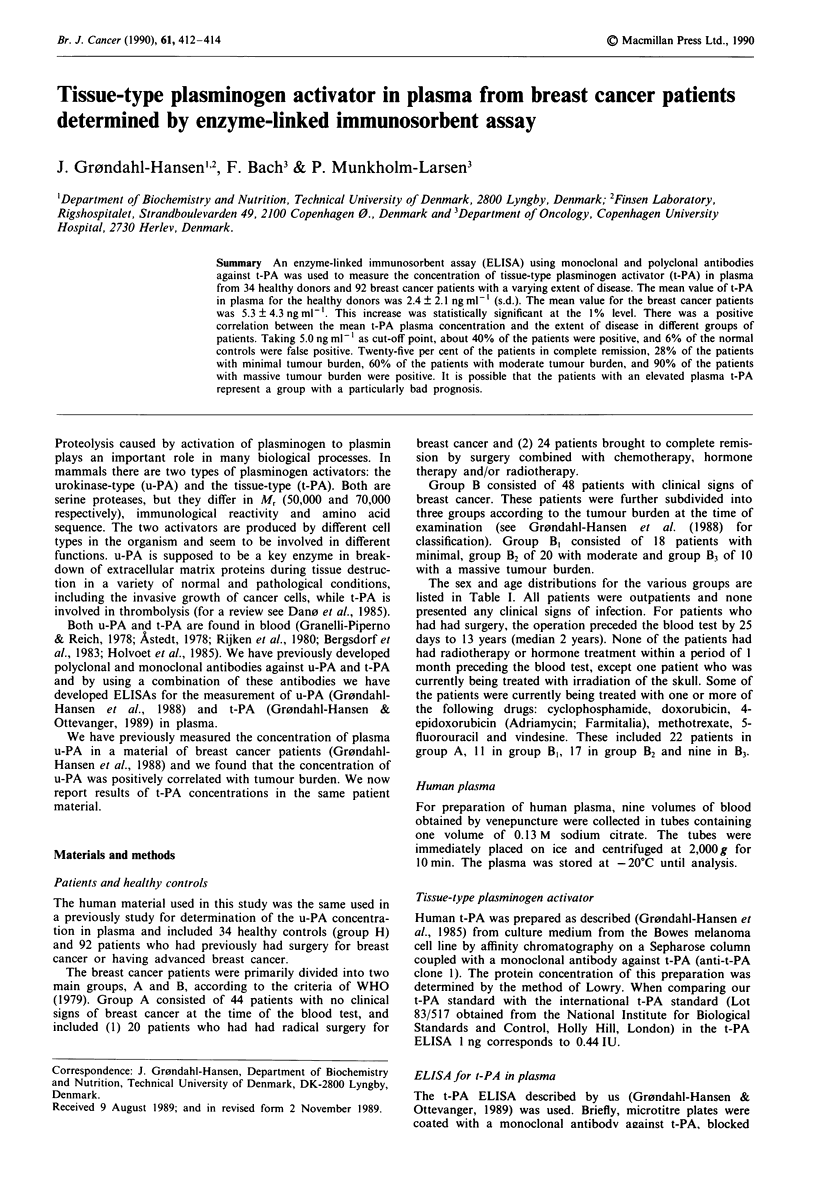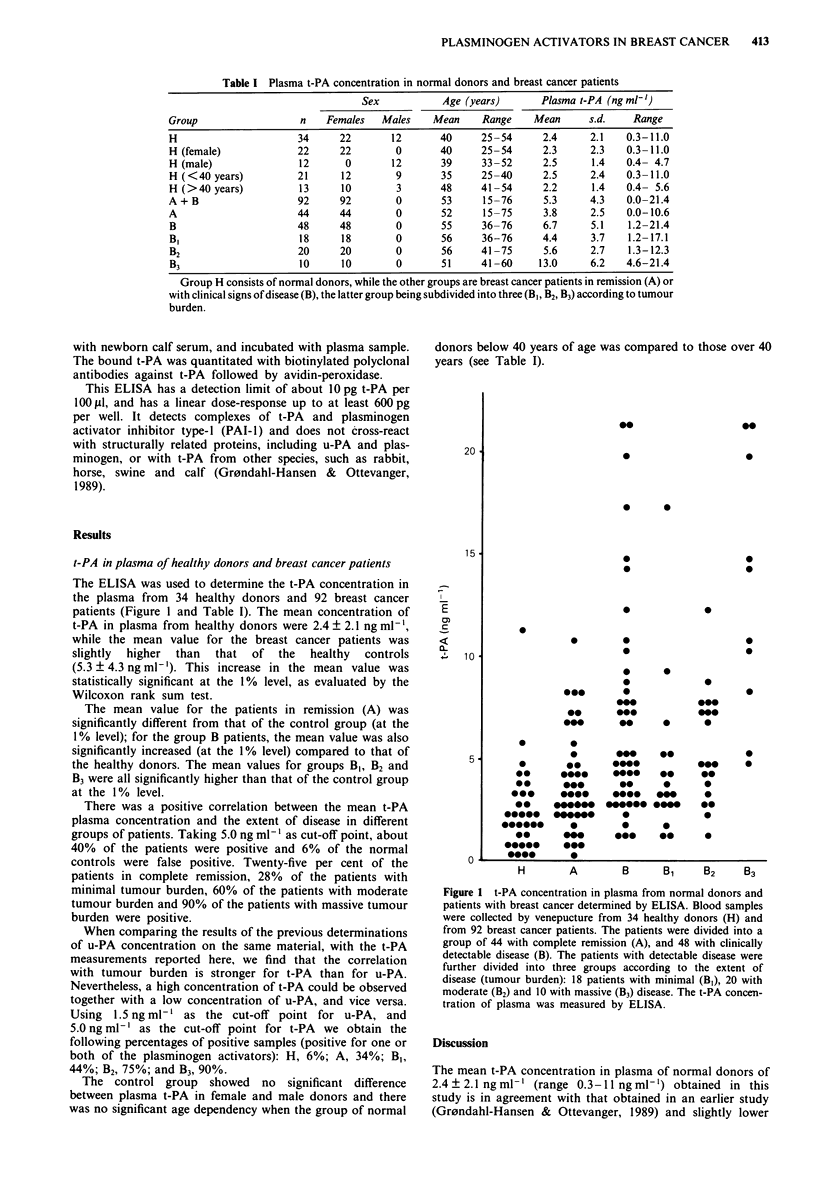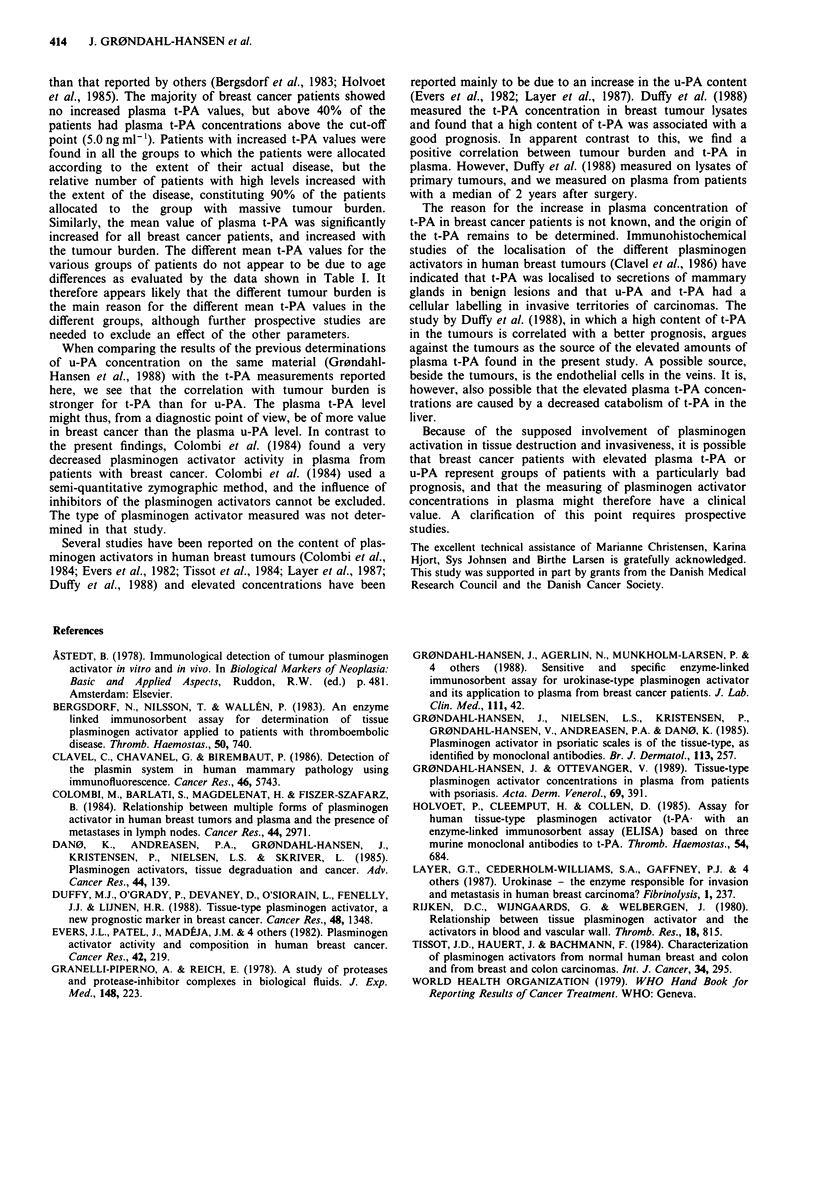# Tissue-type plasminogen activator in plasma from breast cancer patients determined by enzyme-linked immunosorbent assay.

**DOI:** 10.1038/bjc.1990.90

**Published:** 1990-03

**Authors:** J. Grøndahl-Hansen, F. Bach, P. Munkholm-Larsen

**Affiliations:** Department of Biochemistry and Nutrition, Technical University of Denmark, Lyngby.

## Abstract

An enzyme-linked immunosorbent assay (ELISA) using monoclonal and polyclonal antibodies against t-PA was used to measure the concentration of tissue-type plasminogen activator (t-PA) in plasma from 34 healthy donors and 92 breast cancer patients with a varying extent of disease. The mean value of t-PA in plasma for the healthy donors was 2.4 +/- 2.1 ng ml-1 (s.d.). The mean value for the breast cancer patients was 5.3 +/- 4.3 ng ml-1. This increase was statistically significant at the 1% level. There was a positive correlation between the mean t-PA plasma concentration and the extent of disease in different groups of patients. Taking 5.0 ng ml-1 as cut-off point, about 40% of the patients were positive, and 6% of the normal controls were false positive. Twenty-five per cent of the patients in complete remission, 28% of the patients with minimal tumour burden, 60% of the patients with moderate tumour burden, and 90% of the patients with massive tumour burden were positive. It is possible that the patients with an elevated plasma t-PA represent a group with a particularly bad prognosis.


Br. J. Cancer (1990), 61, 412–414 © Macmillan Press Ltd., 1990

### Tissue-type plasminogen activator in plasma from breast cancer patients determined by enzyme-linked immunosorbent assay

J. Grøndahl-Hansen[1,2], F. Bach[3] & P. Munkholm-Larsen[3]

[1]*Department of Biochemistry and Nutrition, Technical University of Denmark, 2800 Lyngby, Denmark;* [2]*Finsen Laboratory, Rigshospitalet, Strandboulevarden 49, 2100 Copenhagen Ø., Denmark and* [3]*Department of Oncology, Copenhagen University Hospital, 2730 Herlev, Denmark.*

**Summary** An enzyme-linked immunosorbent assay (ELISA) using monoclonal and polyclonal antibodies against t-PA was used to measure the concentration of tissue-type plasminogen activator (t-PA) in plasma from 34 healthy donors and 92 breast cancer patients with a varying extent of disease. The mean value of t-PA in plasma for the healthy donors was $2.4 \pm 2.1$ ng ml$^{-1}$ (s.d.). The mean value for the breast cancer patients was $5.3 \pm 4.3$ ng ml$^{-1}$. This increase was statistically significant at the 1% level. There was a positive correlation between the mean t-PA plasma concentration and the extent of disease in different groups of patients. Taking 5.0 ng ml$^{-1}$ as cut-off point, about 40% of the patients were positive, and 6% of the normal controls were false positive. Twenty-five per cent of the patients in complete remission, 28% of the patients with minimal tumour burden, 60% of the patients with moderate tumour burden, and 90% of the patients with massive tumour burden were positive. It is possible that the patients with an elevated plasma t-PA represent a group with a particularly bad prognosis.

Proteolysis caused by activation of plasminogen to plasmin plays an important role in many biological processes. In mammals there are two types of plasminogen activators: the urokinase-type (u-PA) and the tissue-type (t-PA). Both are serine proteases, but they differ in $M_r$ (50,000 and 70,000 respectively), immunological reactivity and amino acid sequence. The two activators are produced by different cell types in the organism and seem to be involved in different functions. u-PA is supposed to be a key enzyme in breakdown of extracellular matrix proteins during tissue destruction in a variety of normal and pathological conditions, including the invasive growth of cancer cells, while t-PA is involved in thrombolysis (for a review see Danø *et al.*, 1985).

Both u-PA and t-PA are found in blood (Granelli-Piperno & Reich, 1978; Åstedt, 1978; Rijken *et al.*, 1980; Bergsdorf *et al.*, 1983; Holvoet *et al.*, 1985). We have previously developed polyclonal and monoclonal antibodies against u-PA and t-PA and by using a combination of these antibodies we have developed ELISAs for the measurement of u-PA (Grøndahl-Hansen *et al.*, 1988) and t-PA (Grøndahl-Hansen & Ottevanger, 1989) in plasma.

We have previously measured the concentration of plasma u-PA in a material of breast cancer patients (Grøndahl-Hansen *et al.*, 1988) and we found that the concentration of u-PA was positively correlated with tumour burden. We now report results of t-PA concentrations in the same patient material.

### Materials and methods

### *Patients and healthy controls*

The human material used in this study was the same used in a previously study for determination of the u-PA concentration in plasma and included 34 healthy controls (group H) and 92 patients who had previously had surgery for breast cancer or having advanced breast cancer.

The breast cancer patients were primarily divided into two main groups, A and B, according to the criteria of WHO (1979). Group A consisted of 44 patients with no clinical signs of breast cancer at the time of the blood test, and included (1) 20 patients who had had radical surgery for breast cancer and (2) 24 patients brought to complete remission by surgery combined with chemotherapy, hormone therapy and/or radiotherapy.

Group B consisted of 48 patients with clinical signs of breast cancer. These patients were further subdivided into three groups according to the tumour burden at the time of examination (see Grøndahl-Hansen *et al.* (1988) for classification). Group $B_1$ consisted of 18 patients with minimal, group $B_2$ of 20 with moderate and group $B_3$ of 10 with a massive tumour burden.

The sex and age distributions for the various groups are listed in Table I. All patients were outpatients and none presented any clinical signs of infection. For patients who had had surgery, the operation preceded the blood test by 25 days to 13 years (median 2 years). None of the patients had had radiotherapy or hormone treatment within a period of 1 month preceding the blood test, except one patient who was currently being treated with irradiation of the skull. Some of the patients were currently being treated with one or more of the following drugs: cyclophosphamide, doxorubicin, 4-epidoxorubicin (Adriamycin; Farmitalia), methotrexate, 5-fluorouracil and vindesine. These included 22 patients in group A, 11 in group $B_1$, 17 in group $B_2$ and nine in $B_3$.

### *Human plasma*

For preparation of human plasma, nine volumes of blood obtained by venepuncture were collected in tubes containing one volume of 0.13 M sodium citrate. The tubes were immediately placed on ice and centrifuged at 2,000 ***g*** for 10 min. The plasma was stored at −20°C until analysis.

### *Tissue-type plasminogen activator*

Human t-PA was prepared as described (Grøndahl-Hansen *et al.*, 1985) from culture medium from the Bowes melanoma cell line by affinity chromatography on a Sepharose column coupled with a monoclonal antibody against t-PA (anti-t-PA clone 1). The protein concentration of this preparation was determined by the method of Lowry. When comparing our t-PA standard with the international t-PA standard (Lot 83/517 obtained from the National Institute for Biological Standards and Control, Holly Hill, London) in the t-PA ELISA 1 ng corresponds to 0.44 IU.

### *ELISA for t-PA in plasma*

The t-PA ELISA described by us (Grøndahl-Hansen & Ottevanger, 1989) was used. Briefly, microtitre plates were coated with a monoclonal antibody against t-PA, blocked

Correspondence: J. Grøndahl-Hansen, Department of Biochemistry and Nutrition, Technical University of Denmark, DK-2800 Lyngby, Denmark.
Received 9 August 1989; and in revised form 2 November 1989.

**Table I** Plasma t-PA concentration in normal donors and breast cancer patients

| | | *Sex* | | *Age (years)* | | *Plasma t-PA (ng ml⁻¹)* | | |
|---|---|---|---|---|---|---|---|---|
| *Group* | *n* | *Females* | *Males* | *Mean* | *Range* | *Mean* | *s.d.* | *Range* |
| H | 34 | 22 | 12 | 40 | 25–54 | 2.4 | 2.1 | 0.3–11.0 |
| H (female) | 22 | 22 | 0 | 40 | 25–54 | 2.3 | 2.3 | 0.3–11.0 |
| H (male) | 12 | 0 | 12 | 39 | 33–52 | 2.5 | 1.4 | 0.4– 4.7 |
| H (<40 years) | 21 | 12 | 9 | 35 | 25–40 | 2.5 | 2.4 | 0.3–11.0 |
| H (>40 years) | 13 | 10 | 3 | 48 | 41–54 | 2.2 | 1.4 | 0.4– 5.6 |
| A + B | 92 | 92 | 0 | 53 | 15–76 | 5.3 | 4.3 | 0.0–21.4 |
| A | 44 | 44 | 0 | 52 | 15–75 | 3.8 | 2.5 | 0.0–10.6 |
| B | 48 | 48 | 0 | 55 | 36–76 | 6.7 | 5.1 | 1.2–21.4 |
| $B_1$ | 18 | 18 | 0 | 56 | 36–76 | 4.4 | 3.7 | 1.2–17.1 |
| $B_2$ | 20 | 20 | 0 | 56 | 41–75 | 5.6 | 2.7 | 1.3–12.3 |
| $B_3$ | 10 | 10 | 0 | 51 | 41–60 | 13.0 | 6.2 | 4.6–21.4 |

Group H consists of normal donors, while the other groups are breast cancer patients in remission (A) or with clinical signs of disease (B), the latter group being subdivided into three ($B_1$, $B_2$, $B_3$) according to tumour burden.

with newborn calf serum, and incubated with plasma sample. The bound t-PA was quantitated with biotinylated polyclonal antibodies against t-PA followed by avidin-peroxidase.

This ELISA has a detection limit of about 10 pg t-PA per 100 µl, and has a linear dose-response up to at least 600 pg per well. It detects complexes of t-PA and plasminogen activator inhibitor type-1 (PAI-1) and does not cross-react with structurally related proteins, including u-PA and plasminogen, or with t-PA from other species, such as rabbit, horse, swine and calf (Grøndahl-Hansen & Ottevanger, 1989).

### Results

### *t-PA in plasma of healthy donors and breast cancer patients*

The ELISA was used to determine the t-PA concentration in the plasma from 34 healthy donors and 92 breast cancer patients (Figure 1 and Table I). The mean concentration of t-PA in plasma from healthy donors were $2.4 \pm 2.1$ ng ml$^{-1}$, while the mean value for the breast cancer patients was slightly higher than that of the healthy controls ($5.3 \pm 4.3$ ng ml$^{-1}$). This increase in the mean value was statistically significant at the 1% level, as evaluated by the Wilcoxon rank sum test.

The mean value for the patients in remission (A) was significantly different from that of the control group (at the 1% level); for the group B patients, the mean value was also significantly increased (at the 1% level) compared to that of the healthy donors. The mean values for groups $B_1$, $B_2$ and $B_3$ were all significantly higher than that of the control group at the 1% level.

There was a positive correlation between the mean t-PA plasma concentration and the extent of disease in different groups of patients. Taking 5.0 ng ml$^{-1}$ as cut-off point, about 40% of the patients were positive and 6% of the normal controls were false positive. Twenty-five per cent of the patients in complete remission, 28% of the patients with minimal tumour burden, 60% of the patients with moderate tumour burden and 90% of the patients with massive tumour burden were positive.

When comparing the results of the previous determinations of u-PA concentration on the same material, with the t-PA measurements reported here, we find that the correlation with tumour burden is stronger for t-PA than for u-PA. Nevertheless, a high concentration of t-PA could be observed together with a low concentration of u-PA, and vice versa. Using 1.5 ng ml$^{-1}$ as the cut-off point for u-PA, and 5.0 ng ml$^{-1}$ as the cut-off point for t-PA we obtain the following percentages of positive samples (positive for one or both of the plasminogen activators): H, 6%; A, 34%; $B_1$, 44%; $B_2$, 75%; and $B_3$, 90%.

The control group showed no significant difference between plasma t-PA in female and male donors and there was no significant age dependency when the group of normal donors below 40 years of age was compared to those over 40 years (see Table I).

**Figure 1** t-PA concentration in plasma from normal donors and patients with breast cancer determined by ELISA. Blood samples were collected by venepucture from 34 healthy donors (H) and from 92 breast cancer patients. The patients were divided into a group of 44 with complete remission (A), and 48 with clinically detectable disease (B). The patients with detectable disease were further divided into three groups according to the extent of disease (tumour burden): 18 patients with minimal ($B_1$), 20 with moderate ($B_2$) and 10 with massive ($B_3$) disease. The t-PA concentration of plasma was measured by ELISA.

### Discussion

The mean t-PA concentration in plasma of normal donors of $2.4 \pm 2.1$ ng ml$^{-1}$ (range 0.3–11 ng ml$^{-1}$) obtained in this study is in agreement with that obtained in an earlier study (Grøndahl-Hansen & Ottevanger, 1989) and slightly lower than that reported by others (Bergsdorf *et al.*, 1983; Holvoet *et al.*, 1985). The majority of breast cancer patients showed no increased plasma t-PA values, but above 40% of the patients had plasma t-PA concentrations above the cut-off point (5.0 ng $ml^{-1}$). Patients with increased t-PA values were found in all the groups to which the patients were allocated according to the extent of their actual disease, but the relative number of patients with high levels increased with the extent of the disease, constituting 90% of the patients allocated to the group with massive tumour burden. Similarly, the mean value of plasma t-PA was significantly increased for all breast cancer patients, and increased with the tumour burden. The different mean t-PA values for the various groups of patients do not appear to be due to age differences as evaluated by the data shown in Table I. It therefore appears likely that the different tumour burden is the main reason for the different mean t-PA values in the different groups, although further prospective studies are needed to exclude an effect of the other parameters.

When comparing the results of the previous determinations of u-PA concentration on the same material (Grøndahl-Hansen *et al.*, 1988) with the t-PA measurements reported here, we see that the correlation with tumour burden is stronger for t-PA than for u-PA. The plasma t-PA level might thus, from a diagnostic point of view, be of more value in breast cancer than the plasma u-PA level. In contrast to the present findings, Colombi *et al.* (1984) found a very decreased plasminogen activator activity in plasma from patients with breast cancer. Colombi *et al.* (1984) used a semi-quantitative zymographic method, and the influence of inhibitors of the plasminogen activators cannot be excluded. The type of plasminogen activator measured was not determined in that study.

Several studies have been reported on the content of plasminogen activators in human breast tumours (Colombi *et al.*, 1984; Evers *et al.*, 1982; Tissot *et al.*, 1984; Layer *et al.*, 1987; Duffy *et al.*, 1988) and elevated concentrations have been reported mainly to be due to an increase in the u-PA content (Evers *et al.*, 1982; Layer *et al.*, 1987). Duffy *et al.* (1988) measured the t-PA concentration in breast tumour lysates and found that a high content of t-PA was associated with a good prognosis. In apparent contrast to this, we find a positive correlation between tumour burden and t-PA in plasma. However, Duffy *et al.* (1988) measured on lysates of primary tumours, and we measured on plasma from patients with a median of 2 years after surgery.

The reason for the increase in plasma concentration of t-PA in breast cancer patients is not known, and the origin of the t-PA remains to be determined. Immunohistochemical studies of the localisation of the different plasminogen activators in human breast tumours (Clavel *et al.*, 1986) have indicated that t-PA was localised to secretions of mammary glands in benign lesions and that u-PA and t-PA had a cellular labelling in invasive territories of carcinomas. The study by Duffy *et al.* (1988), in which a high content of t-PA in the tumours is correlated with a better prognosis, argues against the tumours as the source of the elevated amounts of plasma t-PA found in the present study. A possible source, beside the tumours, is the endothelial cells in the veins. It is, however, also possible that the elevated plasma t-PA concentrations are caused by a decreased catabolism of t-PA in the liver.

Because of the supposed involvement of plasminogen activation in tissue destruction and invasiveness, it is possible that breast cancer patients with elevated plasma t-PA or u-PA represent groups of patients with a particularly bad prognosis, and that the measuring of plasminogen activator concentrations in plasma might therefore have a clinical value. A clarification of this point requires prospective studies.

The excellent technical assistance of Marianne Christensen, Karina Hjort, Sys Johnsen and Birthe Larsen is gratefully acknowledged. This study was supported in part by grants from the Danish Medical Research Council and the Danish Cancer Society.

### References

ÅSTEDT, B. (1978). Immunological detection of tumour plasminogen activator *in vitro* and *in vivo*. In *Biological Markers of Neoplasia: Basic and Applied Aspects*, Ruddon, R.W. (ed.) p. 481. Amsterdam: Elsevier.

BERGSDORF, N., NILSSON, T. & WALLÉN, P. (1983). An enzyme linked immunosorbent assay for determination of tissue plasminogen activator applied to patients with thromboembolic disease. *Thromb. Haemostas.*, **50**, 740.

CLAVEL, C., CHAVANEL, G. & BIREMBAUT, P. (1986). Detection of the plasmin system in human mammary pathology using immunofluorescence. *Cancer Res.*, **46**, 5743.

COLOMBI, M., BARLATI, S., MAGDELENAT, H. & FISZER-SZAFARZ, B. (1984). Relationship between multiple forms of plasminogen activator in human breast tumors and plasma and the presence of metastases in lymph nodes. *Cancer Res.*, **44**, 2971.

DANØ, K., ANDREASEN, P.A., GRØNDAHL-HANSEN, J., KRISTENSEN, P., NIELSEN, L.S. & SKRIVER, L. (1985). Plasminogen activators, tissue degraduation and cancer. *Adv. Cancer Res.*, **44**, 139.

DUFFY, M.J., O'GRADY, P., DEVANEY, D., O'SIORAIN, L., FENELLY, J.J. & LIJNEN, H.R. (1988). Tissue-type plasminogen activator, a new prognostic marker in breast cancer. *Cancer Res.*, **48**, 1348.

EVERS, J.L., PATEL, J., MADÉJA, J.M. & 4 others (1982). Plasminogen activator activity and composition in human breast cancer. *Cancer Res.*, **42**, 219.

GRANELLI-PIPERNO, A. & REICH, E. (1978). A study of proteases and protease-inhibitor complexes in biological fluids. *J. Exp. Med.*, **148**, 223.

GRØNDAHL-HANSEN, J., AGERLIN, N., MUNKHOLM-LARSEN, P. & 4 others (1988). Sensitive and specific enzyme-linked immunosorbent assay for urokinase-type plasminogen activator and its application to plasma from breast cancer patients. *J. Lab. Clin. Med.*, **111**, 42.

GRØNDAHL-HANSEN, J., NIELSEN, L.S., KRISTENSEN, P., GRØNDAHL-HANSEN, V., ANDREASEN, P.A. & DANØ, K. (1985). Plasminogen activator in psoriatic scales is of the tissue-type, as identified by monoclonal antibodies. *Br. J. Dermatol.*, **113**, 257.

GRØNDAHL-HANSEN, J. & OTTEVANGER, V. (1989). Tissue-type plasminogen activator concentrations in plasma from patients with psoriasis. *Acta. Derm. Venerol.*, **69**, 391.

HOLVOET, P., CLEEMPUT, H. & COLLEN, D. (1985). Assay for human tissue-type plasminogen activator (t-PA· with an enzyme-linked immunosorbent assay (ELISA) based on three murine monoclonal antibodies to t-PA. *Thromb. Haemostas.*, **54**, 684.

LAYER, G.T., CEDERHOLM-WILLIAMS, S.A., GAFFNEY, P.J. & 4 others (1987). Urokinase – the enzyme responsible for invasion and metastasis in human breast carcinoma? *Fibrinolysis*, **1**, 237.

RIJKEN, D.C., WIJNGAARDS, G. & WELBERGEN, J. (1980). Relationship between tissue plasminogen activator and the activators in blood and vascular wall. *Thromb. Res.*, **18**, 815.

TISSOT, J.D., HAUERT, J. & BACHMANN, F. (1984). Characterization of plasminogen activators from normal human breast and colon and from breast and colon carcinomas. *Int. J. Cancer*, **34**, 295.

WORLD HEALTH ORGANIZATION (1979). *WHO Hand Book for Reporting Results of Cancer Treatment*. WHO: Geneva.